# Automating the Illumina DNA library preparation kit for whole genome sequencing applications on the flowbot ONE liquid handler robot

**DOI:** 10.1038/s41598-024-58963-2

**Published:** 2024-04-08

**Authors:** Erin Meijers, Fabienne B. Verhees, Dennis Heemskerk, Els Wessels, Eric C. J. Claas, Stefan A. Boers

**Affiliations:** Department of Medical Microbiology, Leiden University Medical Center, Albinusdreef 2, 2333 ZA Leiden, The Netherlands

**Keywords:** Next-generation sequencing, Microbiology

## Abstract

Whole-genome sequencing (WGS) is currently making its transition from research tool into routine (clinical) diagnostic practice. The workflow for WGS includes the highly labor-intensive library preparations (LP), one of the most critical steps in the WGS procedure. Here, we describe the automation of the LP on the flowbot ONE robot to minimize the risk of human error and reduce hands-on time (HOT). For this, the robot was equipped, programmed, and optimized to perform the Illumina DNA Prep automatically. Results obtained from 16 LP that were performed both manually and automatically showed comparable library DNA yields (median of 1.5-fold difference), similar assembly quality values, and 100% concordance on the final core genome multilocus sequence typing results. In addition, reproducibility of results was confirmed by re-processing eight of the 16 LPs using the automated workflow. With the automated workflow, the HOT was reduced to 25 min compared to the 125 min needed when performing eight LPs using the manual workflow. The turn-around time was 170 and 200 min for the automated and manual workflow, respectively. In summary, the automated workflow on the flowbot ONE generates consistent results in terms of reliability and reproducibility, while significantly reducing HOT as compared to manual LP.

## Introduction

Over the past decade, developments in next-generation sequencing (NGS) methods have provided a wealth of genetic information in the form of whole genome sequences. Sequencing large genomes, such as human, plant, or animal genomes, provided valuable information for disease research and population genetics^1–3^, whereas microbial whole-genome sequencing (WGS) has had widespread use in the fields of clinical, environmental, industrial, and veterinary microbiology^4–7^. Specifically, WGS has been increasingly applied in clinical microbiological laboratories to aid in microbial identification and genotyping of (pathogenic) microorganisms for epidemiological purposes, as well as detection of antimicrobial resistance genes, virulence factors, and/or plasmids^4,8,9^.

The WGS workflow contains four basic steps: nucleic acid (NA) extraction, library preparation (LP), sequencing, and data analysis. LP represents one of the most labor-intensive step of the whole workflow, as sequencing is performed on automated NGS-platforms and subsequent data analyses often is using automated bioinformatic pipelines^10–12^. NA extraction can either be performed on fully automated NA-extraction platforms or manually, depending on the sample type and microbial species under investigation^12^.

One of the many LP kits on the marked today is the Illumina DNA Prep (Illumina, San Diego, CA, USA). This kit uses on-bead tagmentation chemistry to fragment DNA and add (index) adapter sequences by reduced-cycle PCR amplification. Amplified libraries are then purified and pooled before starting an NGS-run. Although the Illumina DNA Prep kit is being widely used for many different applications, the protocol includes many time-consuming, repetitive pipetting steps and is prone to errors when performed manually. These limitations can be overcome by automating LP steps using robotic liquid-handlers^13^.

Here, we describe the development and evaluation of an automated protocol of the Illumina DNA Prep using the flowbot ONE liquid-handler (Flow Robotics, Copenhagen, Denmark). The performance of the automated protocol was evaluated by processing 16 bacterial strains both manually and automatically, after which the library DNA yields and NGS results obtained from both workflows were compared. Eight of these 16 bacterial strains were processed twice in two independent flowbot ONE runs to assess reproducibility of results. In addition, the turn-around time (TAT) and hands-on-time (HOT) of both workflows were compared.

## Methods

### Bacterial strains and DNA extraction

All bacterial strains were obtained from the routine diagnostic laboratory at the Leiden University Medical Center (LUMC) in the Netherlands. A total of 16 bacterial strains were selected for this study covering a range of Gram-positive and Gram-negative species, including *Staphylococcus aureus* (4x)*, Clostridioides difficile* (3x), *Klebsiella pneumoniae* (2x), *Pseudomonas aeruginosa* (2x), *Acinetobacter baumannii* (1x)*, Enterobacter cloacae* (1x), *Enterococcus faecalis* (1x)*, Escherichia coli* (1x), and *Stenotrophomonas maltophilia* (1x)*.* The species of the bacterial strains was determined using matrix-assisted laser desorption/ionization time-of-flight (MALDI-TOF) mass spectrometry^14^. DNA was extracted from a 0.5 McF suspension of the bacterial strains using the automated QIAsymphony SP platform (Qiagen, Hilden, Germany) or the manual QIAamp DNA Blood Mini Kit (Qiagen) both following the manufacturer’s recommendations, after which the DNA concentrations were measured using a Qubit fluorometer (Thermo Fisher Scientific, Waltham, MA, USA) to determine the input volume for the LP. The bacterial strains were not traceable to individual patients, omitting the need for approval by an ethical committee.

### Illumina DNA Prep

Sequencing libraries were prepared manually from 20 ng of purified DNA extracts, using the Illumina DNA Prep in combination with the Illumina DNA/RNA UD Indexes Sets A-D (Illumina). All four main steps of the LP protocol (i.e., tagmentation, post-tagmentation clean-up, PCR amplification, and post-amplification clean-up) were performed according to the manufacturer’s instructions.

The manual LP protocol was translated into the automated protocol using the flowbot ONE software v1.03.06a (Flow Robotics). The installation file of this protocol is available from Flow Robotics upon request.

After performing manual and automated LPs, the DNA concentration of each library was measured using the Qubit fluorometer to compare library DNA yields between workflows and to prepare equimolar pools for NGS.

### Next-generation sequencing and data analysis

Pooled sequencing libraries were bidirectionally sequenced using the MiniSeq platform with 2 × 150 bp chemistry (Illumina). To correct for technical variation due to uneven sequencing depths and potential bias introduced by the manual pooling of libraries before sequencing, the total number of reads per library was normalized to one million using seqtk (v1.4, https://github.com/lh3/seqtk). Subsampled reads were assembled de novo with Velvet (v1.1.04) as part of the SeqSphere + version 8.3.5 software package (Ridom Bioinformatics GmbH, Münster, Germany), after which the quality of the assemblies was evaluated per library by investigating the average sequence read depths, the number of contigs, and the contig N50 values. In addition, 14 of the 16 bacterial strains were genotyped using species-specific core genome multilocus sequencing typing (cgMLST) schemes that was also performed with SeqSphere + software. Each scheme consisted of a fixed number of target genes where mutations in these genes resulted in a specific complex type (CT). The cgMLST schemes used in this study consisted of 1861, 2147, 2358, 3867, 2390, 1972, and 2513 target genes for *S. aureus*^15^, *C. difficile*^16^, *K. pneumoniae*^17,18^*, P. aeruginosa*^19^*, A. baumannii*^20^*, E. faecalis*^21^*,* and *E. coli*^17,22,23^*,* respectively. No cgMLST schemes were available for genotyping *E. cloacae* and *S. maltophilia* strains.

## Results

**Figure 1:**
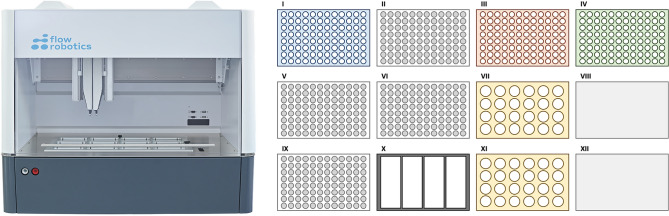
Schematic view of the flowbot ONE (left) and the deck set-up used for automation of the Illumina DNA Prep (right). Both the pre-PCR and post-PCR parts of the workflow use the same deck set-up, with I: 96-well coldplate compatible with 8-tube strips (shown in blue); II: filter tips (1–50 μl, shown in white); III: 96-well plate containing small-volume Illumina DNA Prep reagents (shown in orange); IV: magnetic deck compatible with 96-well plates (shown in green); V: filter tips (2–200 μl, shown in white); VI: filter tips (1–50 μl, shown in white); VII: 24-tube rack containing medium-volume Illumina DNA Prep reagents (shown in yellow); IX: filter tips (2–200 μl, shown in white); X: Four column reservoir containing large-volume Illumina DNA Prep reagents (shown in black); XI: 24-tube rack containing the final, sequence-ready library DNA (shown in yellow). Deck positions VIII and XII are not used for the automation of the Illumina DNA Prep protocol (shown in solid grey).

The flowbot ONE liquid-handler is designed as a multi-purpose pipetting instrument. For the automation of the Illumina DNA Prep protocol, the robot was equipped with a 1-channel 2–200 µl pipetting module, as well as an 8-channel 2–200 µl pipetting module, and the 12-position deck was set up with a heating/cooling device, a magnetic device, and several modules for placement of pipetting tips and tube racks (Fig. 1). The workflow was split into pre-PCR and post-PCR, requiring the user to transfer pipetted PCR tube strips to and from the PCR machine. The Illumina DNA/RNA UD indexes are added manually to each LP before starting the PCR program to minimize potential negative effects of freeze-thawing cycles of these indexes between runs. Using this automated workflow, a total of eight samples were processed per flowbot ONE-run, while the maximum throughput of this set-up is 24 samples per run.

The performance of the automated workflow was assessed by comparing the library DNA yields obtained with the automated workflow to those obtained with the manual workflow. For this, a total of 16 bacterial DNA extracts, derived from a wide range of diverse bacterial species, were processed using both workflows. As shown in Table 1, an increase in DNA concentration was measured after performing each LP. Both workflows performed equally well as the DNA yields obtained were comparable, with a median of a 1.5-fold difference between both workflows. There was one outlier involving a *K. pneumoniae* LP that yielded 1.9 ng/ul with the automated workflow, while the DNA concentration obtained after the manual workflow was 10.9 ng/µl. In addition, the automated workflow yielded reproducible library DNA concentrations for eight LPs that were processed twice from the same DNA extract, with a maximum difference of 2 ng/µl between a replicate LP. The lowest library DNA yield obtained with the automated workflow was 0.6 ng/µl, which was still sufficient to start an NGS-run, as the final loading concentration for the MiniSeq platform is 1.2 pM.

**Table 1:** Comparison of DNA concentrations measured after DNA extraction and after performing the manual and automated LP workflows.

ID	Species (Gram-stain)	DNA concentrations (ng/µl)			Factor difference^b^
		DNA extract	Manual LP	Automated LP^a^	
1	*A. baumannii* (-)	0.8	15.2	7.6	2.0
2	*S. aureus* ( +)	0.1	2.9	1.7	1.7
3	*S. aureus* ( +)	0.2	7.7	5.2	1.5
4	*P. aeruginosa* (-)	1.3	15.1	10.4	1.5
5	*K. pneumoniae* (-)	0.4	10.9	1.9	5.6
6	*E. faecalis* ( +)	< 0.1	1.4	0.6	2.2
7	*S. maltophilia* (-)	1.2	16.9	9.4	1.8
8	*C. difficile* ( +)	1.2	16.5	13.0	1.3
9	*E. cloacae* (-)	0.5	11.6	11.4 13.1	1.0 0.9
10	*P. aeruginosa* (-)	2.4	6.9	7.1 6.3	1.0 1.1
11	*K. pneumoniae* (-)	0.5	9.0	12.0 11.4	0.8 0.8
12	*E. coli* (-)	0.7	15.8	10.3 11.7	1.5 1.4
13	*S. aureus* ( +)	0.3	16.0	9.3 8.3	1.7 1.9
14	*C. difficile* ( +)	1.2	11.7	18.1 19.1	0.6 0.6
15	*C. difficile* ( +)	1.7	17.6	16.1 14.1	1.1 1.2
16	*S. aureus* ( +)	1.1	13.2	8.7 9.4	1.5 1.4

*LP* library preparation, − Gram-negative bacteria, +  Gram-positive bacteria.

^a^The automated LPs were performed in three independent flowbot ONE runs, with sample 1–8 processed in the first run and samples 9–16 processed in the second and third run for reproducibility testing.

^b^The factor differences were calculated by dividing the DNA concentrations measured after manual LP by the DNA concentrations of the libraries obtained with the automated workflow.

All 40 DNA libraries were sequenced in nine multiplex NGS-runs to evaluate the quality of sequence data obtained using both LP workflows. Sequence data was normalized to one million sequence reads per library, after which the reads were assembled into genome contigs. As shown in Table 2, each of the 40 assemblies consisted of an average sequence read depth of at least 32 reads, a maximum of 536 contigs, and a contig N50 value of at least 65,117 nucleotides, indicating good quality genome assemblies^24,25^. The quality of the genome assemblies obtained with the automated workflow compared to the quality of the genome assemblies obtained with the manual workflow showed a median value of only 1.0, 1.1, and 0.9-fold differences between the average read depth, number of contigs, and contig N50 values, respectively. Moreover, the quality of the genome assemblies of the eight libraries that were prepped twice in two independent flowbot ONE-runs were comparable (with a maximum of a 1.2, 1.6, and 1.1-fold difference for average read depth, number of contigs, and contig N50 values, respectively), indicating a good reproducibility of results. Finally, 14 of the 16 bacterial strains were genotyped using cgMLST and showed 100% concordance between manually and automatically prepped libraries (Table 2). No cgMLST-scheme was available for the remaining two bacterial strains.

**Table 2:** Comparison of NGS-results obtained from the DNA libraries prepped with the manual and automated workflows.

ID	Species	Manual LP				Automated LP^a^			
		Average read depth	No. of contigs	Contig N50 value	CT	Average read depth	No. of contigs	Contig N50 value	CT
1	*A. baumannii*	65	122	130,855	5739	56	130	120,793	5739
2	*S. aureus*	53	93	148,765	10,250	69	57	175,655	10,250
3	*S. aureus*	44	107	87,218	29,549	71	71	236,617	29,549
4	*P. aeruginosa*	35	127	253,249	3485	37	125	194,842	3485
5	*K. pneumoniae*	44	124	249,794	9468	44	127	208,058	9468
6	*E. faecalis*	82	73	252,526	3279	67	84	171,989	3279
7	*S. maltophilia*	54	62	404,192	NA	54	74	307,186	NA
8	*C. difficile*	51	191	153,433	6412	47	191	153,389	6412
9	*E. cloacae*	37 –	145 –	123,665 –	NA –	50 44	57 56	406,176 406,176	NA NA
10	*P. aeruginosa*	41 –	60 –	457,320 –	3881 –	39 40	69 63	378,423 391,655	3881 3881
11	*K. pneumoniae*	46 –	101 –	315,903 –	9559 –	35 41	161 98	259,608 335,928	9559 9559
12	*E. coli*	42 –	281 –	92,775 –	24,800 –	44 37	260 292	106,206 106,221	24,800 24,800
13	*S. aureus*	46 –	71 –	157,271 –	20,040 –	69 58	53 45	200,036 181,835	20,040 20,040
14	*C. difficile*	32 –	241 –	65,117 –	6044 –	44 41	127 140	149,052 188,829	6044 6044
15	*C. difficile*	50 –	533 –	118,000 –	6759 –	46 47	536 412	112,835 97,762	6759 6759
16	*S. aureus*	50 –	108 –	108,892 –	34,349 –	56 59	68 58	170,114 196,683	34,349 34,349

*CT* complex type, *LP* library preparation, *NA* not available.

^a^The automated LPs were performed in three independent flowbot ONE runs, with sample 1–8 processed in the first run and samples 9–16 processed in the second and third run for reproducibility testing.

**Figure 2:**
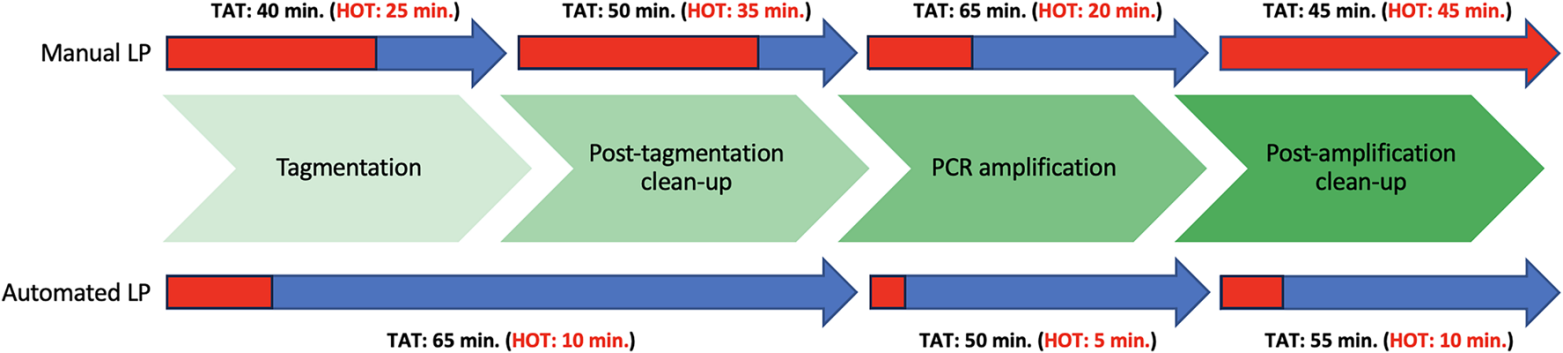
Schematic view of the turn-around time (TAT) and the hands-on time (HOT) required to process eight samples using the manual and automated Illumina DNA Prep workflows. The green blocks represent the different steps that are part of the Illumina DNA Prep kit. The HOT is shown in red and is part of the TAT that is shown in blue.

One of the main goals of automating the Illumina DNA Library Prep protocol was to reduce HOT. As shown in Fig. 2, the automated workflow shortened the HOT required for processing eight samples to 25 min compared to the 125 min needed when processing the same eight samples using the manual workflow. The TAT per eight samples was 170 and 200 min for the automated and manual workflow, respectively.

## Discussion

Whole genome sequencing (WGS) provides the most comprehensive data about any given species and has the potential to become a dominant technology used in the diagnostic clinical microbiology laboratories for infection control or detailed identification of pathogens^26^. The implementation of WGS in routine diagnostics will however require several adaptations in the laboratory workflow. In particular, LPs represent one of the most critical steps in the WGS workflow but are also the most labor-intensive steps that are prone to human error when performed manually^13,27^. Therefore, we converted the manual protocol of the widely used Illumina DNA Library Prep kit to an automated protocol on a multipurpose robot, the flowbot ONE.

The flowbot ONE is available in most markets globally, through distributors or by contacting Flow Robotics directly. The robot is fully modular in terms of configuration, protocol design and generic consumables that can be used. This contrasts with highly sophisticated platforms such as Hamilton and Tecan robots that are only compatible with their own (expensive) system-specific consumables, which eventually can become a limitation^13^. In addition, while these advanced robots can deliver fully automated, walk-away LPs (including on-board PCR), they have a capacity that is often too large for the average microbial WGS project performed in diagnostic clinical microbiology laboratories. Therefore, the flowbot ONE offers a cost-effective solution, by providing semi-automated workflows (excluding on-board PCR) with a capacity that is more suited for small-scale WGS applications. The same applies to the OT-2 open-source programming robot from Opentrons, with the added advantage that the OT-2 does offer on-board PCR for fully automated LP workflows. However, Opentrons did not provide service-level agreements in Europe at the time of investigation. Lacking service-level agreements in Europe makes the implementation of these robots in ISO-15189 accredited laboratories difficult. From May 2024, in-house developed in-vitro diagnostic devices may only be used in European laboratories that are compliant with EN ISO 15189 and attention is also needed to fulfill the other requirements of the In Vitro Diagnostic Regulation (IVDR) in a timely manner^28^.

The Illumina DNA Prep kit is one of many LP kits on the market today. Specific LP kits are available for the different sequencing techniques used in laboratories, including NGS (e.g., Illumina sequencing) and third-generation sequencing such as PacBio and nanopore sequencing technologies. Although these LP kits have their own specific protocols, they all have in common that they consist of many (repetitive) pipetting steps that can be easily automated using multipurpose robots such as the flowbot ONE. In addition, most of these LP kits (including the Illumina DNA Prep) make use of magnetic beads in different steps of their protocols. The strength of the magnetic deck, together with the ability to pipette supernatant without disturbing the magnetic beads is therefore of great importance for generating high-quality DNA libraries. In this study, the Armadillo semi-skirted 96-well plates (Thermo Fisher Scientific, Waltham, MA, USA) were implemented into the protocol as these plates facilitated a strong magnetic force between Illumina magnetic beads and the magnetic deck from Flow Robotics, where our routine diagnostic 96-well PCR plates (Bio-Rad, Hercules, CA, USA) did not (data not shown).

The automated workflow resulted in high and reproducible library DNA yields that were comparable to those obtained with the manual workflow. No dropouts were encountered for the 24 LP performed with the automated workflow as well as for the 16 manually performed LPs. In addition, after NGS, similar assembly quality values were obtained from the normalized number of reads for each of the 40 individual DNA libraries. All 40 assemblies met our routine diagnostic quality criteria for WGS, which requires a minimum depth of 30 sequence reads and contains < 1000 contigs with a contig N50 value of > 15,000 nucleotides per assembly. The high-quality sequence data obtained using both manual and automated workflows was further confirmed with a 100% agreement of the final NGS-result (i.e., cgMLST CT).

The current automated workflow is designed to process samples in batches of eight. Without changing the described deck set-up and equipped pipetting modules, throughput can be easily expanded to process 24 samples per run with no increase in HOT by adjusting the settings in the flowbot ONE software. Higher throughput LP workflows are also feasible but require adjustments to the robot’s deck-layout. Using the automated workflow, an excess of reagents equaling four samples was used to reduce the chance on aspirating air bubbles towards the end of a pipetting run. This slightly increases the cost of library prepping using the automated workflow compared to the manual workflow, as the manual workflow requires an excess of reagents equaling three samples when processing the same eight samples simultaneously.

With the automated workflow, the TAT to process eight samples was comparable to the TAT using the manual workflow. However, the HOT for processing eight samples was reduced to 25 min for the automated workflow, while the manual workflow takes 125 min to process the same sample size. This significant reduction is due to all the heavy repetitive pipetting performed by the flowbot ONE instead of a laboratory technician, which also minimized the chance of human error.

In summary, the newly developed and validated automated LP workflow on the flowbot ONE generates robust and high-quality DNA libraries. Although we have designed this automated workflow to prepare DNA libraries for small-scale microbial WGS experiments, the easy-to-use software of the flowbot ONE allows automation of most LP protocols available for the many NGS and third-generation sequencing applications without the need of advanced programming skills. Automating these critical LP steps will further reduce sequencing costs (by reducing HOT) while delivering consistent results in terms of reliability and reproducibility.

## Data Availability

The installation file of the automated Illumina DNA Prep workflow is available from Flow Robotics upon request. The sequence data generated and analysed during the current study can be accessed online at https://www.ncbi.nlm.nih.gov/bioproject/PRJNA1064853, accession number PRJNA1064853.
